# The diagnosis and treatment of 56 cases of breast hamartoma: a single-center analysis and a review of the literature

**DOI:** 10.3389/fmed.2024.1494768

**Published:** 2025-01-22

**Authors:** Hao Su, Caiyun Bai, Zhimin Fan, Di Wu, Fengjiang Qu

**Affiliations:** ^1^Department of Breast Surgery, General Surgery Center, First Hospital of Jilin University, Changchun, China; ^2^Department of Emergency Surgery, First Hospital of Jilin University, Changchun, China

**Keywords:** breast hamartoma, misdiagnosis, treatment, ultrasound imaging, pathological analysis

## Abstract

**Introduction:**

To analyze and summarize the clinical and pathological features of breast hamartoma to enhance clinicians’ awareness of this rare and benign lesion of the breast.

**Methods:**

We retrospectively described the clinical data, imaging results, and pathological findings of 56 patients with breast hamartoma who underwent surgical treatment at the First Hospital of Jilin University between January 2005 and December 2020, and summarized the features. Additionally, a literature review was conducted using the PubMed database for clinical reports on breast hamartoma and analyzed them.

**Results:**

This study included 56 female patients. Preoperative ultrasound revealed round or elliptical heterogeneous echoes with clear boundaries: hypoechoic in 35 cases (63%), iso-echoic in 8 cases (14%), hyperechoic in 1 case (2%), mixed echo in 9 cases (16%), slightly strong echo in 1 case (2%), and uneven echo of fat in 2 cases (4%). Mammography was performed in 33 cases, revealing clear and dense shadows in 20 cases (61%) and dense shadows on the outer edges in 16 cases (48%). The excised masses were solid, with light yellow and gray cut surfaces. Pathological analysis identified ducts and fibrous tissue, with intra-tumoral fat content ranging from 10 to 90%.

**Conclusion:**

Breast hamartoma is prevalent among perimenopausal women and is characterized by ultrasound and radiography; surgical intervention remains the primary treatment with a good prognosis.

## Introduction

Breast hamartoma is a rare and benign disease of the breast. Hogeman and Osbtang were the first to describe this lesion in 1968 (1). Subsequently, the disease was named breast hamartoma by Arrigoni et al. (2). Due to its low incidence, clinicians often have insufficient knowledge of breast hamartoma, leading to frequent misdiagnoses. Previous studies were mostly presented in the form of case studies; few large-scale systematic studies have been undertaken. In this study, we describe the clinical diagnosis and treatment data for 56 patients with breast hamartoma, confirmed through surgical treatment and pathological examination, from January 1, 2005, to December 31, 2020, in the Department of Breast Surgery at the First Hospital of Jilin University. The data were retrospectively analyzed and discussed to improve the efficacy of diagnosis and treatment for this disease.

## Materials and methods

We retrospectively collected and analyzed clinical diagnosis and treatment data from 56 patients diagnosed with breast hamartoma, confirmed through surgical treatment and pathological examination, at the Department of Breast Surgery, First Hospital of Jilin University, from January 1, 2005, to December 31, 2020. The analysis encompassed the basic clinical characteristics, imaging examination results, surgical data, and pathological findings of these patients. Additionally, we conducted a comprehensive search of the PubMed database for literature related to breast hamartoma. Our literature review of PubMed identified a total of 18 reviews of case literature published between 2011 and 2020 (Table 1). This study adheres to a retrospective design, ensuring patient privacy by excluding any identifiable personal data. The study complies with the Declaration of Helsinki and relevant laws and regulations, waiving the need for informed consent from participants, and has received approval from the Ethics Committee of the First Hospital of Jilin University (ethics approval number: 2024–1,072).

**Table 1:** Summary of 18 reviews of breast hamartoma published in the PubMed between 2011 and 2020.

References	Gender (case)	Age (years)	Past history	Size/Location	Ultrasound	X-ray
Mahmoud et al. (3)	F(1)	26	Normal	13 cm/OL	Uneven echo, peripheral isoechoic fat and central fibrous tissue echo	Clear boundaries, different density of mass and fat fiber
Bhatia et al. (4)	F(1)	18	NR	2 cm/ OU	Clear boundary and low echo	Fibroadenomatous image
Phan et al. (8)	M(1)	41	Normal	3.4 cm/OU	Clear boundary Heterogeneous hypoechoic	High density oval mass
Xia et al. (22)	F(2)	Mean 31.5 (28–35)	NR	Mean 4.8 cm (3–6.5)/OU	Heterogeneous hypoechoic	NR
Li et al. (9)	M(1)	30	Normal	2.4 cm/OU	NR	Well defined mixed density
Fox and Devanathan (15)	F(1)	15	NR	4 cm/IU	Irregular hypoechoic	NR
Panagopoulos et al. (24)	F(1)	44	NR	5.7 cm/NR	Heterogeneous hypoechoic	Translucent, clear boundary
Ghaedi and Howlett (12)	F(1)	45	NR	10 cm/OU	Echo attenuation area	Mixed fat/soft tissue density，central opaque dense area
Elhence et al. (20)	F(1)	35	NR	12 cm/OU	NR	Uneven fibrous gland tissue and scattered high density, Small amount of calcification in the high transparent area
Amir and Sheikh (5)	F(13) M(1)	Mean 33 (18–51)	NR	Mean 3.8 cm (1–9.5)/O	Clear boundary hyperechoic or heteroechoic in inner part	Heterogeneous dense nodules, Radiopaque pseudocapsule encircling
Wang and He (11)	F(1)	41	Normal	11 cm/OU	Heterogeneous inhomogeneous echo	Well defined, mixed density
Makiguchi et al. (13)	F(1)	40	NR	18 cm/NR	Inhomogeneous isoechoic and hypoechoic	NR
Sevim et al. (6)	F(27)	Mean 41.8	NR	Mean 3.9 cm/NR	Inhomogeneous internal echo pattern	Fat that can pass through rays; all kinds of fibrous and glandular tissues have smooth edges
Tosuner et al. (16)	F(2)	Mean 43.5	NR	Mean 6.0 cm/OU	Inhomogeneous echo/isoecho	Clear boundary
Nangia et al. (25)	F(1)	48	Normal	10 cm/OU	Clear outline, mixed calcification echo	NR
Vergine et al. (27)	F(1)	30	Normal	5.5 cm/L	Hypoechoic	NR
Nasit et al. (26)	F(1)	45	NR	9 cm/OU	NR	Clear boundary, light transmission around, moderately radiopaque in center
Mizuta et al. (28)	F(1)	38	NR	2.8 cm/OU	Clear boundary and low echo	Clear boundary and equidensity
Current case (2021)	F(56)	Mean 42.43	Table 3	Median 4.0 cm/Table 3	Heterogeneous echo	Clear and dense boundary shadow wrapped by impermeable rays, transparent shadow with fat

F, female; M, male; NR, no reported; OL, outer lower; OU, outer upper; IU, inner upper; O, outer.

## Results

All 56 cases were female with a mean age of 42.43 ± 1.58 years (range: 19–75 years), with a medical history lasting from 1 day to 17 years. Among these, 49 cases had palpable masses on physical examination. Seven cases were detected by routine imaging examinations but were negative on physical examinations, presenting no pain, nipple discharge, or other accompanying symptoms. Of the 49 cases, 27 were on the left side (one with multiple occurrences on the left side) and 22 were on the right side; the median diameter of a single tumor was 4.0 cm (range: 1.5–12 cm). The specific past history and examination data are shown in Tables 2, 3.

**Table 2:** Physical examination characteristics of 49 cases of palpable masses.

	Number of cases	(%)
Texture		
Hard	5	10%
Soft	7	14%
Tenacity	37	76%
Boundary		
Clear	42	86%
No-clear	7	14%
Form		
Regular	40	82%
Irregular	9	18%
Activity		
Good	45	92%
Difference	4	8%

**Table 3:** The past history and tumor information of 56 patients.

	Number of cases	%
Location		
Left	31	55%
Right	25	45%
Quadrant		
Outside	24	43%
Inside	10	18%
Other	22	39%
Past history		
Gynecological surgery	2	3%
Thyroid surgery	8	15%
Hppendix operation	6	10%
Hypertension	5	9%
Healthy	36	62%
Breast surgery	1	1%
Ultrasound	56	100%
BI-RADS category		
II	2	3.6%
III	47	84.0%
IV	7	12.5%
Echoic		
Hypoechoic	35	62.5%
Iso-echoic	8	14.3%
Hyperechoic	1	1.8%
Mixed echo	9	16.1%
Strong echo	1	1.8%
Uneven echo	2	3.6%
Mammography	33	100%
BI-RADS category		
I	6	18.2%
II	2	6.1%
III	18	54.5%
IV	7	21.2%
Shadow		
Negative	6	18.2%
Asymmetric	1	3.0%
Punctate calcification	2	6.1%
High	15	45.5%
Isodensity	6	18.2%
Low	2	6.1%
Mixed	1	3.0%
Border		
Clear	20	60.6%
Unclear	4	12.1%
Morphology		
Regular	21	63.6%
Irregular	3	9.1%

**Figure 1:**
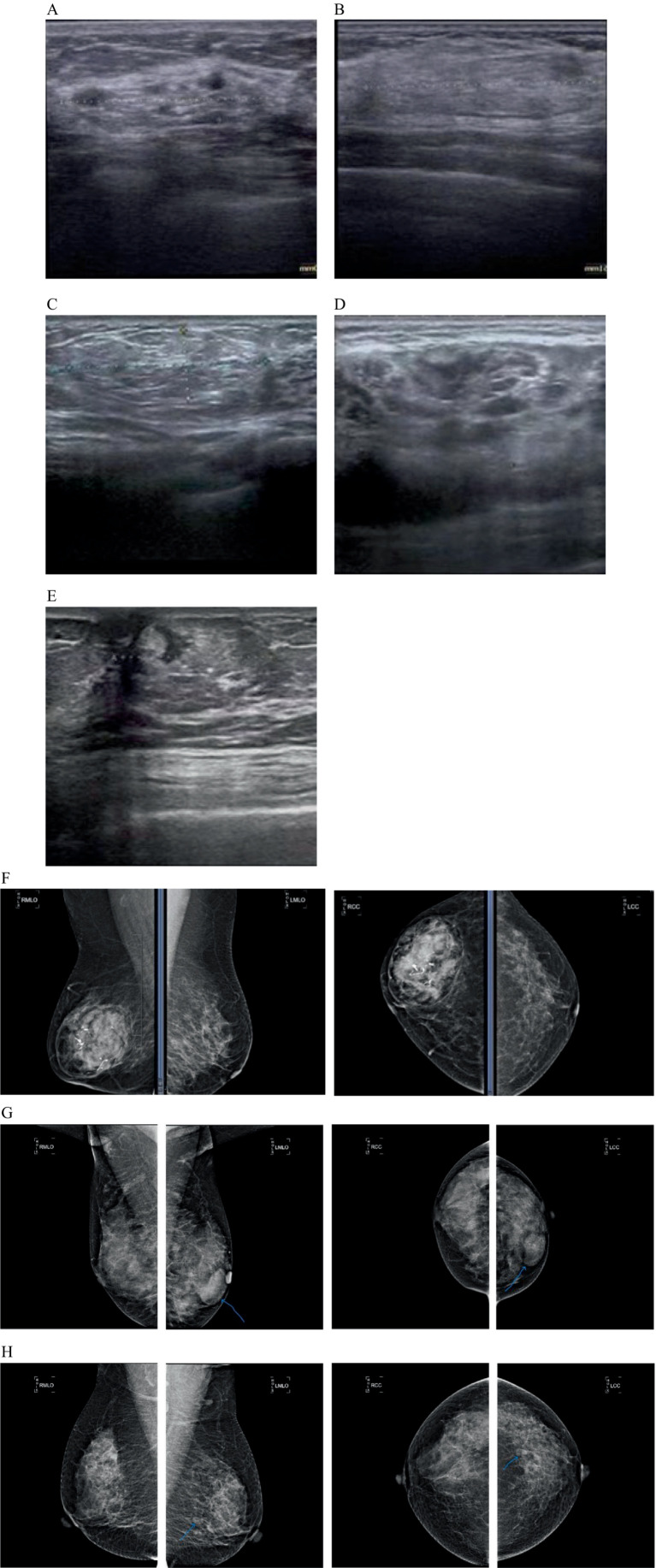
The characteristics of breast hamartoma on color Doppler ultrasound and mammography (breast X-ray imaging). **(A)** Ultrasound image showing a mixed echo mass. **(B)** Ultrasound image showing an iso-echo mass. **(C)** Ultrasound image showing an uneven echo mass of fat. **(D)** Ultrasound image showing a hypoechoic echo mass. **(E)** Ultrasound image showing a slightly strong echo mass. **(F)** Mammogram showing an oval mass of high, equal and low mixed density with clear margin and coarse calcification in the right breast. **(G)** Mammogram showing multiple iso-density mass in the left breast, the larger one is in the inner lower quadrant, about 4.0×2.6 cm in size, the boundary is clear. **(H)** Mammogram showing round-like low-density shadows in the lower outer quadrant of the left breast.

All patients underwent preoperative ultrasound examination (Figures 1A–E). Tumors were hypoechoic in 35 cases, iso-echoic in eight cases, and hyperechoic in one case; in addition, a mixed echo was evident in nine cases, a slightly strong echo in one case, and an uneven echo of fat in two cases. The mean length of a single tumor (from the 55 cases measured by ultrasound) was 3.59 ± 0.26 cm (range: 0.9–9.7 cm). The diameters of the multiple tumors on the left side were 1.1 cm and 1.3 cm, respectively. All tumors were round or oval. A partition was seen in the tumor of one case, and calcification was seen in the tumor of another case. Color Doppler Flow Imaging (CDFI) revealed a small amount of blood flow signal in the tumors of four cases, and a small amount of blood flow signal around the tumor in one case. No obvious abnormal lymph nodes were detected in the bilateral axilla. There were two cases of BI-RADS Category II, 47 cases of Category III, and seven cases of Category IV.

Preoperative breast mammography examinations were performed in 33 cases (Figures 1F–H). Of these, six cases had negative mammography results, one case showed an focal asymmetry, two cases showed punctate calcification, 15 cases showed a high-density, six cases showed an isodensity, two cases showed low-density, and one case showed mixed-density. Of the 20 cases with clear borders, four had fuzzy, inadequate, or unclear boundaries; 21 had regular morphology, and three had insufficient or irregular morphology. In 24 cases, the molybdenum target measured a mean length and diameter of 4.0 ± 3.8 cm. In 16 cases, a halo sign was observed at the periphery of the mass opacity. The study included 2 cases categorized as BI-RADS Category 2, 18 cases as Category 3, and 7 cases as Category 4.

**Figure 2:**
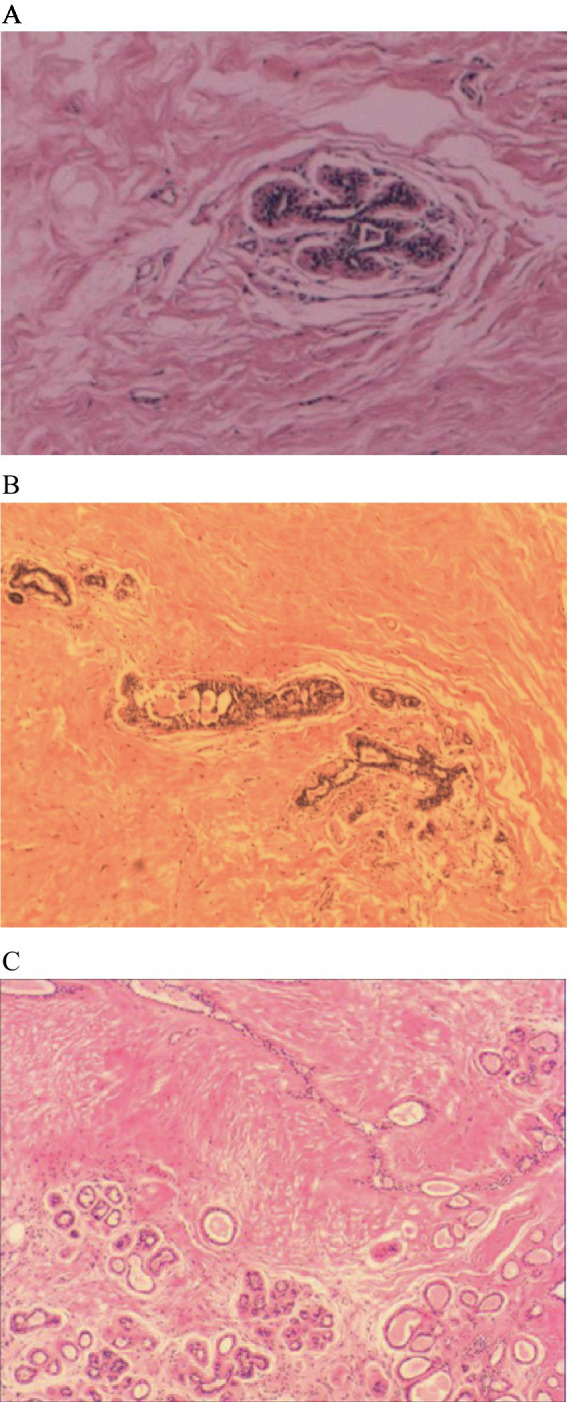
The histopathological features of breast hamartoma on paraffin-embedded sections (H&E staining). **(A)** Pathological section:hematoxylin and eosin stained mammary glandular tissue (magnification, ×10). **(B)** Pathological section:hematoxylin and eosin stained mammary glandular tissue (magnification, ×10). **(C)** Pathological section:hematoxylin and eosin stained mammary glandular tissue (magnification, ×10).

All 56 patients underwent surgical treatment. Vacuum-assisted breast biopsy (VABB) or corresponding segmental resection of breast lesions was performed according to the specific condition of the tumor. With regards to the masses, all were solid lesions, and the cut surface was mainly pale yellow and gray-white, or single gray and pale yellow. Intraoperative rapid frozen sections were prepared for 16 cases: 11 cases were indicative of benign lesions or fibroadenoma, three cases were indicative of hamartoma, and two cases were indicative of adenopathy. Postoperative paraffin pathological analysis revealed that all cases were consistent with hamartoma; ducts and fibrous tissues were visible, and the content ranged from 10 to 90%. Capsules were evident in specimens undergoing resection of the corresponding section of breast lesions (Figures 2A–C).

In this article, we summarized the diagnosis and our treatment experience of 56 cases of breast hamartoma. This is a retrospective analysis with the largest number of cases to date; our data verified many conclusions in the existing literature. For perimenopausal women and occasional painless breast masses, ultrasound shows regular and heterogeneous echoes, and mammography shows clear and dense shadows surrounded by radiopaque borders, the possibility of hamartoma should be considered. These are widely considered to be benign lesions, but active surgical treatment may be beneficial for some cases. The final diagnosis should be combined with the results of pathological examinations.

## Discussion

Breast hamartoma is a rare benign tumor that includes fibers and fat in component ratios that are different from those of normal breast tissue. The incidence of breast hamartoma ranges from 0.7 to 4.8% of all benign breast tumors (3–7) and can occur in both males and females, although the incidence in males is more uncommon than in females (5, 8, 9). In females, breast hamartoma can occur in the late lactation and premenopausal stages, but is most commonly detected in premenopausal women (7, 10); the age of onset is usually 40–45 years (4, 5, 11). The mean age of our case series was 42.43 ± 1.58 years, and premenopausal cases accounted for 71% of all patients. Upon clinical examination, hamartoma is usually a single mass with a clear boundary, regular morphology, soft to hard texture, and no tenderness (5, 8, 9). Tumor size varies significantly, usually between 2 and 5 cm (7). Some studies have reported huge breast hamartomas with a diameter greater than 10 cm (3, 11–13). In our study cohort, except for one case with multiple hamartomas on the left side, the other hamartomas were single, with a median diameter of 4.0 cm (range: 1–12 cm). The lesions were mostly located in the outer quadrant; this is because the outer quadrant contains more breast tissue than the inner quadrant. There were no obvious tumors upon breast examination in seven patients, thus accounting for approximately 12.5% of cases in this group. All tumors were detected by physical examination and related imaging examinations. The mean tumor diameter was 1.29 cm, as measured by ultrasound. Of these, three cases underwent mammography. Due to a 0.5 cm diameter high-density shadow, punctate calcification, and negative findings, it has been reported that almost 60% of breast hamartomas are impalpable (5). Our present results suggest that a tumor with a diameter of approximately 1 cm is difficult to palpate during physical examinations; therefore, it is vital that women who meet these conditions should undergo regular screening for breast disease. The literature describes few patients with a history of disease. Our present research found that in addition to patients who were healthy in the past, a proportion of patients had undergone surgery for thyroid disease. The thyroid and breasts are both target organs for hormonal stimulation. A previous study by Jaklic et al. (14) proved that estrogen and progesterone receptors are expressed in diseased thyroid tissues. The retrospective analyses published by Amir and Sheikh (5) and Sevim et al. (6) also reported that breast hamartomas express estrogen and progesterone receptors; thus, female steroid hormones are related to the development of hamartoma. Moreover, estrogen, progesterone, and thyroid hormone are also known to have synergistic effects with each other, although there is a lack of knowledge with regards to the mechanisms that underlie this synergy or whether these mechanisms are related to hamartomas; this requires further research.

Due to the density of breast glands, ultrasound is currently the preferred imaging procedure for screening breast diseases in our country. Ultrasound images of hamartomas can appear as oval or quasi-circular masses with clear boundaries; this depends on the amount of fat and fibroglandular components and presents as hypoechoic or mixed echoes. The internal echoes are uneven and some are visible. These tumors are surrounded by a pseudo-capsule and a few lesions may have punctate blood flow signals (9–11, 15, 16). Consistent with existing literature, we found that the echo type and uniformity differed according to different proportions of components in the lesions, Georgian-Smith et al.’s study is consistent with our findings, where the majority of lesions presented as hypoechoic nodules (17). In some studies, hamartomas predominantly exhibit mixed echogenicity (6, 18).These discrepancies underscore the variability in the imaging manifestations of hamartomas, which is likely influenced by a multitude of factors including the composition of internal tissues, imaging techniques, clinical context, and pathological characteristics. For instance, hamartomas at different stages may exhibit varying echogenic features; the fat within the hamartomas may atrophy over time, with an increase in fibrous components, leading to a more heterogeneous echo pattern on imaging; seven cases showed an irregular shape, unclear borders, or echoes in the ipsilateral armpit and enlarged lymph nodes, thus suggesting BI-RADS category IV. This serves as a reminder that there are a few cases for which ultrasound imaging can detect potentially malignant tumors.

Some researchers consider that the first choice for breast hamartoma imaging is mammography, which manifests as a well-defined oval or flat mass, with a clear and dense border of fibrous tissue and adenoid components, containing dense fatty tissue, often surrounding the lesion. There is also a thin envelope (8, 16, 19). The proportions of hamartoma components are disordered; therefore, these lesions exhibit different manifestations in terms of density effects (3, 5, 6, 8, 20); this tendency was also evident in the current study. Over recent years, evidence has gradually increased to show that hamartomas exhibit specific imaging characteristics and that imaging is a useful diagnostic tool. Therefore, mammography is still necessary. Some patients have expressed concern about the potential impact of X-ray radiation; this should be fully explained to increase compliance.

Breast hamartoma can be cured by surgical resection; this remains the currently accepted treatment for hamartomas. Many patients who require surgery for benign breast tumors and desire better cosmetic outcomes benefit from the popularization and promotion of vacuum-assisted breast biopsy (VABB). This technique is suitable for benign breast tumors, fibroadenomas, nodules, asymmetric density, multifocal lesions, and microcalcifications with surgical indications less than or equal to 2 cm (21). For patients who meet the indications of VABB, we normally provide patients with local infiltration anesthesia, make an incision of about 0.5 cm close to the tumor, and completely remove the tumor under ultrasound guidance. For all other patients, we make an appropriately sized incision at the body surface projection of the tumor or along the areola edge according to the size of the tumor, and perform segmentectomy under general anesthesia. In the present study, a combination of preoperative clinical data and related examinations indicated that all cases should be considered as benign tumors and met the appropriate indications for complete VABB surgery; two patients had large tumors and had cosmetic requirements. Most of the tumors were removed by minimally invasive techniques, while the remaining tumors were treated with a small incision followed by segmental resection. The remaining patients underwent segmental resection of breast lesions. In all cases, the surgical procedure was smooth, and postoperative recovery was good and in line with the requirements of rapid rehabilitation surgery.

Breast hamartoma is considered to be the result of dysplasia, defined as a random combination of lobular breast tissue, fibrous tissue, and adipose tissue (22) but with a clear boundary with normal breast tissue. In our study, there were 51 cases involving intraoperative histological rapid frozen sections; of these, 15 cases (29%) were diagnosed correctly and 29 cases (57%) were initially considered, by surgical experience, as benign lesions or fibroadenomas. The main limitation of diagnosis is related to the experience of the pathology department; if a hamartoma contains a significant amount of fibrous gland tissue, it can often be misdiagnosed as a fibroadenoma. Therefore, the final diagnosis depends on paraffin pathology. In general, most hamartomas are lobulated, solid, and the cut surface is pale yellow or off-white. Histologically, hamartomas exhibit different proportions of breast ducts, breast lobules, fibrous tissue, and adipose tissue (5, 16). Research has shown that 99% of hamartomas contain a certain proportion of adipose tissue, most commonly found in the interstitium of hamartomas (4). The distribution of fat and lobules is the main characteristic that distinguishes hamartomas from fibroadenomas (12). Pathologists do not elaborate on the classification criteria of hamartomas. Previously, some researchers further divided hamartomas into three subtypes according to different component ratios: fibroadenolipoma, myoid hamartoma, and chondolilipoma (11, 23). Histochemistry plays a key role in subtype classification. The existing literature provides certain descriptions of myoid hamartomas, thus providing us with a description of the main characteristics of breast myoid hamartoma for the first time; these characteristics included smooth muscle actin, vimentin and binding protein diffuse positive staining. These myoid hamartomas have a similar HMGA2 rearrangement as lipomas and grow on mutant mesenchymal stem cells, which can differentiate into stromal cells, adipocytes and smooth muscle cells (22, 24–27).

At present, breast hamartoma is considered to be a benign lesion, although invasive malignant tumors may develop (28, 29). Baer et al. (30) reported the first case of HER2(+) invasive ductal carcinoma originating from cases with breast hamartomas and reviewed the reports of 19 known cases of cancerous hamartomas. The mean size of hamartomas with invasive ductal carcinoma (IDC) was reported to be 6.0 cm (range: 1.5–16.7 cm), and most cases have suspicious manifestations of calcification, nodules and asymmetry on mammograms; comprehensive recommendations for hamartomas larger than 6.0 cm with imaging abnormalities should undergo needle biopsy (19). There is no definite treatment guideline for cancer caused by hamartoma. However, it is evident that malignant tumors should be treated in accordance with surgical methods, and that adjuvant treatment should be considered. The specific relationship between breast hamartomas and malignant tumors is still under discussion.

## Data Availability

The original contributions presented in the study are included in the article/supplementary material, further inquiries can be directed to the corresponding authors.
